# How sleep and fatigue shape statements in evidence: A psycho-legal perspective

**DOI:** 10.3389/fcogn.2024.1423413

**Published:** 2024-06-25

**Authors:** Zlatan Krizan, Breanna Curran

**Affiliations:** Department of Psychology, Iowa State University, Ames, IA, United States

**Keywords:** sleep, fatigue, confessions, voluntariness, eyewitness, admissibility, testimony

## Abstract

Testimonial evidence in the form of verbal accounts by victims, witnesses, and suspects plays a critical role in investigations and judicial proceedings, often serving as the only evidence during a trial. The psychological nature of testimonies causes this form of evidence to be inherently limited, motivating psycho-legal scholars to identify both risk factors and solutions necessary to improve its reliability. To this end, the current perspective argues that *sleep-related fatigue* is a formative factor that influences the fidelity of statements and confessions provided during legal interactions. Specifically, it considers the prevalence of sleep disruption among subjects interacting with the criminal justice system, its likely impact on memory of victims and witnesses, and the role of sleep deprivation in confessions. In view of legal doctrines relevant to both evidentiary and constitutional considerations, this analysis is meant to motivate future work at the intersection of sleep-related fatigue and legal processes.

## 1 Introduction

Despite advancements in technology that have improved the availability and reliability of physical and documentary evidence (e.g., DNA, electronic communications), verbal accounts provided by suspects, victims, and witnesses remain indispensable in legal proceedings. These accounts play a crucial role in establishing facts, determining culpability, and navigating plea-bargaining agreements (Walton, 2008). However, such statements in evidence, including confessions, originate from fallible human sources. Inherent limitations in cognitive capacity, incomplete knowledge of relevant events, and various psychological biases, all signal the need to understand factors shaping the accuracy of verbal accounts solicited during legal processes (Davis and Loftus, 2012).

Legal psychology has made significant progress with regard to probative value of eyewitness claims, victim accounts, and criminal confessions (Kassin et al., 2010; Wixted and Wells, 2017). Yet, limited attention has been paid to *sleep-related fatigue*—a pervasive and impactful force on human behavior. In response, this article aims to highlight the relevance of sleep-related fatigue for the reliability of statements and confessions obtained during legal proceedings. Critically, sleep-related fatigue is both widespread among subjects interacting with the criminal justice system and consequential for statements made to law-enforcement and testimony given in court. As outlined below, such fatigue stems both from insufficient and poor sleep endemic among populations that interact with law-enforcement (Jackson and Testa, 2022).

## 2 Sleep, fatigue, and behavior

Processes governing sleep-wake behavior are specified by the “2-process” model of sleep-wake regulation (Edgar et al., 1993; Borbely and Achermann, 1999). First, the homeostatic process generates a sleep drive (“S”), analogous to hunger, where sleep pressure rises with prolonged wakefulness, subsequently dissipating upon sleep onset, and rising again upon awakening (Suzuki et al., 2013). Secondly, the circadian process produces cyclical changes in ~24-h periods (“C”), dampening sleep propensity during early evening hours and enhancing it during early morning hours, primarily orchestrated by the central body clock entrained to the light-dark cycle (Hastings et al., 2018). These two processes support consolidation of sleep during the night and wakefulness throughout the day. Nonetheless, east-west travel or persistent nocturnal activity can desynchronize internal circadian rhythms from external light-dark cycles or social demands, leading to circadian misalignment and subsequent difficulties with falling asleep or staying awake when required (Wittmann et al., 2006; Waterhouse et al., 2007). Given the pivotal role sleep plays in maintaining organismic homeostasis and optimal functioning, sleep-circadian disruptions (i.e., insufficient or displaced sleep) precipitate a spectrum of consequences spanning various neurobehavioral domains. Disruptions in sleep and circadian processes result in far-reaching, dose-dependent, and multifaceted consequences for alertness, emotion, and cognitive control (Krizan, 2019; Palmer et al., 2023; Zimmerman et al., 2024).

First, the most salient consequence of disrupted sleep is diminished psychomotor alertness, evident in delayed reactions and lapses in awareness (Lim and Dinges, 2010). These deteriorations are accentuated with prolonged sleep loss and pronounced during nocturnal hours (Van Dongen et al., 2003; Holding et al., 2021). In addition to these deficits in alertness, subjective perceptions of sleepiness do not perfectly track the objective performance deficits, leading to limited insight into impairment following sleep loss (Zhou et al., 2012; Åkerstedt et al., 2014). As a result, subjects interacting with the criminal justice system experiencing the consequences of sleep loss and circadian misalignment (e.g., a suspect undergoing a nightime interrogation) may not be fully aware of their impairment.

Second, sleep disruption exerts a detrimental impact on motivational and emotional functioning, precipitating feelings of fatigue and avoidance of effort. Sleep-deprived individuals exhibit a propensity for less demanding activities and easier performance strategies, pointing to motivational deficits (Massar et al., 2019). Concurrently, alterations in emotional functioning, characterized by diminished positive affect, heightened anxiety, and increased frustration, highlight the harmful impact of sleep disruption on emotional regulation (Krizan and Hisler, 2019; Tomaso et al., 2021). Over time, disrupted sleep is associated with more frequent mental distress and higher risk for depression (Franzen and Buysse, 2008; Blackwelder et al., 2021). Such emotional dysregulation may make individuals more vulnerable to interrogative pressure.

Third, sleep loss has been implicated in reasoning impairments, stemming from the diminished cognitive control and working memory which require effortful attention reliant on prefrontal cortical circuits (Harrison and Horne, 2000a; Honn et al., 2019; Whitney et al., 2023). Deficits in attentional control and memory following sleep disruption manifest as reliance on heuristic processing and diminished propensity for deliberate reasoning (Satterfield et al., 2019). These cognitive impairments are accentuated with increasing sleep debt, suggesting such fatigue-induced deviations from normative reasoning strategies may impact individuals' comprehension of legal risks.

Taken together, we refer to this array of consequences as *sleep-related fatigue*, given the decreased ability of the brain to maintain arousal and sustained engagement with the environment following extended wakefulness (especially at times of low circadian alerting, Chong and Baldwin, 2021). Fatigue in general is a complex phenomenon as it involves passive, active, and sleep-circadian components, as well as underlying physical conditions (Boksem and Tops, 2008; Saxby et al., 2013; Andrillon et al., 2019). Nevertheless, sleep disruption exacerbates all forms of fatigue, although there are substantial individual differences in the magnitude of this disruption and the ability to cope (Veksler and Gunzelmann, 2018; Massar et al., 2019; Hudson et al., 2020). Specifically, individuals can cope somewhat efficiently with low doses of fatigue when given flexibility in how to manage a task or solve a problem, but inevitably show performance decrements following significant sleep loss or when the control they have over a task is limited (Hockey, 2013).

## 3 Sleep-related fatigue among subjects in the criminal justice system

Due to sleep-related fatigue, the prevalence and severity of sleep and circadian disruption among individuals within the criminal justice system warrants careful consideration. In this vein, several large surveys indicate that individuals interacting with law-enforcement often experience poor or inadequate sleep. For example, data from the fragile families and child wellbeing study drawing on 3,444 U.S. youth indicate that those who reported being stopped by the police had higher odds of shorter and more disrupted sleep (Jackson et al., 2020). The number of reported sleep problems was even higher among those with more intense, direct, or intrusive interactions with the law, such that *most* of the individuals who were taken into custody reported at least one sleep problem. A similar large-scale analysis of U.K. adolescents indicated that those with more police contact reported shorter sleep, longer sleep onset, and more frequent mid-sleep awakenings (Jackson and Testa, 2022). These findings emphasize the heightened likelihood of chronic sleep disruption among individuals providing statements to the police, especially those in custody and subject to interrogation (Partin and Lehmann, 2023). Among the incarcerated population the extent of insomnia and sleep disturbances is even higher (Geijsen et al., 2018; Morris et al., 2021); these individuals often provide statements and decisions during plea negotiations or while serving as jailhouse informants.

Victims of crime are especially likely to suffer from poor sleep quality and insufficient sleep. Evidence indicates that victims of physical violence and sexual assault are at higher risk for insomnia, likely exacerbated by traumatic experiences associated with the crime (Steine et al., 2012; Zhang et al., 2019). Analysis of over 14,000 Australians indicated that those who were victims of physical violence within the past year had 16% higher probability of poor sleep quality and reported sleeping 15 min less per night during a typical week (Clark et al., 2019). Even such a minor curtailment accumulates to more than an hour of missed sleep per week. These sleep deficits may be especially severe for some individuals or regions owing to social inequities in sleep opportunities and broader societal changes that undermine sleep (Grandner, 2017; Fang et al., 2021; Caraballo et al., 2022).

Finally, criminal activity often occurs during nighttime hours, requiring police to collect statements from individuals experiencing varying degrees of sleep loss and circadian disruption. For example, the most recent data from the FBI's Uniform Crime Reporting Program (2022) show that nearly a third of crimes against persons occurred during nighttime (10 pm−6 am) [Crime/Law Enforcement Stats (UCR Program)., 2024]. Similarly, survey and daily diary studies of police interviewing practices find that almost 20% of investigative interviews occur at night, suggesting potential impairment among subjects of such interviews (Kassin et al., 2007; Krizan et al., 2023).

In summary, evidence from multiple countries indicates high prevalence of insomnia, insufficient, and poor-quality sleep among individuals interacting with law enforcement, particularly victims and those with more extensive law-enforcement contact. In the next section, we argue that such individuals are especially vulnerable to the adverse effects of sleep-circadian disruption and resultant fatigue, namely compromised reliability and accuracy of their statements across legal interactions.

## 4 Influence of sleep and fatigue on statements in evidence

Alongside physical (e.g., DNA) and documentary evidence (e.g., images), *testimonial* evidence plays a critical role in establishing guilt or innocence of potential suspects (Walton, 2008). Testimonial evidence comes in the form of verbal statements or claims made by individuals, as exemplified by victim accounts of the crime, eyewitness reports, or self-incriminating statements provided by potential suspects. Such evidence is essential and often the only type available (Brewer and Gary, 2011). As a result, it is critical to understand processes that impact its reliability and validity, as well as costs associated with admitting such evidence when it is faulty. By the time a statement is presented in court, it may or may not be a veridical representation of what an individual had actually observed, said, or done, creating difficulties for triers of fact to discern whether testimonial evidence is reliable or sincere (Wixted and Wells, 2017).

### 4.1 Sleep-related fatigue can undermine witness and victim memory

Because any witness statement begins as a memory, the *accuracy* of that memory becomes a critical consideration when evaluating statements. This consideration is very salient in cases relying on eyewitness claims; whether a witness identifies the actual culprit has important consequences both for achieving justice and for protecting the innocent (Agathocleous, 2020). Critically, there is mounting evidence that losing sleep undermines the fidelity of witness recollections. Decades of empirical research have shown that declarative memory performance benefits from sleep both before and after learning, including semantic and episodic information (Ashton et al., 2020; Newbury et al., 2021; Cunningham et al., 2022).

During early investigative stages when the spontaneous recall of witnessed events is paramount (e.g., during a neighborhood canvas), fatigued individuals may fail to recollect or misremember details (Harrison and Horne, 2000b). For example, Thorley (2013) found that reports of current sleepiness and poor sleep the prior night predicted worse recollection of details from a mock crime scene. Carlson et al. (2022) found a similar damaging impact of disrupted sleep on spontaneous recall of details. Analyses focusing on witness accuracy during line-up procedures as a function of sleep loss (occurring later once a suspect is identified by police) have found similar trends, although effects on recognition accuracy were smaller and inconsistent across studies (Stepan et al., 2017; Morgan et al., 2019). Moreover, loss of sleep prior to witnessing an event can increase susceptibility to misinformation, such that individuals are more likely to incorporate subsequent suggestions into their memories (Frenda et al., 2014; Lo et al., 2016).

While more research is needed, existing evidence suggests that sleep disruption undermines free and accurate recall, alongside a more limited impact on recognition tasks like line-up identifications (Carlson et al., 2022). Beyond recently witnessed events, testimonial evidence also involves accounts of more distant past, especially in “cold” (unsolved) cases. Research on autobiographical memory suggests that sleep-deprived individuals recall less information and spatio-temporal details, whilst fatigue may hamper memory search efforts or disrupt memory structures (Zare Khormizi et al., 2019; Ashton et al., 2020; Krizan et al., 2021). In brief, both law enforcement and triers of fact need to be cognizant of potential sleep loss experienced by witnesses prior to engaging in or recollecting relevant events, as this likely undermines the accuracy of their memories (especially timelines and episodic information).

Sleep-related concerns are especially noticeable for accounts provided by victims (including police involved in shootings or traumatic incidents). Traumatic experiences during crimes engender significant stress, benefiting memory for central details (e.g., a weapon pointed at the victim), at the expense of peripheral details (Gagnon and Wagner, 2016; Loetscher and Goldfarb, 2024). Furthermore, sleep following such an incident prioritizes memory consolidation of central and negative details at the cost of neutral or peripheral details (Payne et al., 2012; Denis et al., 2022). As a result, the importance of interviewing victims immediately after the crime, but also following several sleep opportunities, is attracting attention by both victim and law-enforcement organizations (Hope et al., 2016; Campbell, 2022).

### 4.2 Sleep-related fatigue can increase false confessions

Confessions are one of the most influential forms of testimonial evidence. Within the U.S. justice system confessions can be the sole basis for a conviction and are found compelling by jurors (Alceste et al., 2021). However, false confessions do occur and have been implicated in a significant proportion of wrongful convictions [DNA Exonerations in the United States (1989–2020)., 2023] (Innocence Project). Critically, fatigue is a known contributing factor to false confessions. In their analysis of proven false confessions, Leo and Drizin (2008) found that lengthy interrogations were present in the vast majority of cases, averaging 16 h. While the exact timing of interrogations within the 24-h period is not always known, such lengthy interrogations will inevitably extend into nighttime hours unless starting in the morning, necessitating sleep and circadian disruption. In parallel, numerous false confessions have been obtained during early morning hours, as in the case of sailor Danial Williams of the “Norfolk Four” who falsely confessed to committing a gruesome murder between 5 and 6 am following an 11-h interrogation (Woody and Forrest, 2020).

Preliminary experimental evidence supports the premise that sleep deprivation increases false confessions. For example, one study found that sleep deprived individuals were five times more likely to sign a false confession statement for both minor and moderate offenses (Scherr et al., 2014; Frenda et al., 2016). Note that sleep loss could contribute both to compliant (feigned) and internalized (believed) false confessions (Leo and Drizin, 2008; Kassin et al., 2010). First, the intense desire for rest and sleep motivates withdrawal, disengagement, and effort conservation (Engle-Friedman and Young, 2019; Axelsson et al., 2020). These motivations are in direct conflict with the demands of stressful, lengthy, and repetitive questioning characteristic of guilt-presumptive interrogation tactics known to increase false confessions (Madon et al., 2017). As a result, fatigued subjects may superficially comply with interrogators who insist that they admit wrong-doing as a means to escape a fatiguing situation. Second, the cognitive impairments associated with significant sleep loss (and stress) may contribute to internalization of one's guilt. As highlighted earlier, sleep loss engenders significant problems with working memory, attention to the environment, time perception, and cognitive flexibility (Whitney et al., 2023). When combined with false evidence ploys (e.g., “we have your DNA”) or psychological manipulation (e.g., hypnosis), such fatigue could render individuals especially vulnerable to interrogative pressure and confuse them into accepting their own guilt. Impact of fatigue may also be further aggravated by intellectual disability or acute intoxication.

### 4.3 Sleep-related fatigue can impair reasoning about legal consequences

Sleep-related fatigue, specifically the consequences regarding cognition, also carry implications for legal reasoning of individuals interacting with the criminal justice system. For example, suspects undergoing interrogations tend to make short-sighted confession decisions, prioritizing immediate relief from the adverse interrogative situation while ignoring the more severe long-term costs of self-incrimination (e.g., arrest, Madon et al., 2013). In this vein, sleep-deprived subjects are likely to experience their situation as especially averse due to increased distress, which alongside diminished capacity for reasoning likely places them at a higher risk for suboptimal outcomes. For example, very fatigued subjects may waive Miranda rights even when contrary to their self-interest, although research is lacking.

In addition, sleep-related fatigue could impact the plea-bargaining process. As most cases are settled via pleas prior to any trial, undesirable bargains may result when the charged individual negotiates a plea while experiencing sleep-related fatigue. Along these lines, research on betting behavior suggests sleep-deprived individuals become less sensitive to risky options, do not adjust their confidence as they become tired, and suffer more financial losses as a result (Frings, 2012; Fraser et al., 2013; Hamel et al., 2021). Given most individuals considering plea agreements are incarcerated and incarceration is known to induce severe sleep disruption (Morris et al., 2021), sleep-related fatigue deserves more attention as a potential factor during this critical stage of legal decision-making and justice administration.

Accordingly, it is critical to note significance for various legal protections afforded by the U.S. Constitution. For example, the U.S. Supreme Court has ruled that 36 h of continuous interrogation is *per se* coercive as it precludes mental freedom on the part of the suspect as required by the 14th Amendment (Ashcraft v. Tennessee, 1944). Sleep deprivation (akin to intoxication) may further impinge on the individuals' ability to understand their rights under the U.S. Constitution —for example, the ability to provide knowing, intelligent, and voluntary waivers of Miranda rights prior to custodial interrogation (Vuotto and Ciccone, 2006; Mindthoff et al., 2022).

## 5 Discussion

This brief perspective highlighted existing knowledge about the impact of sleep-related fatigue on testimonial evidence. Taken together, the surveyed evidence suggests that sleep-related fatigue threatens the validity of statements critical to the justice system. The most persuasive evidence currently available concerns the negative impact of sleep-related fatigue on the reliability of witness recollections and proclivity to falsely admit wrongdoing when under social pressure, although the extremity of fatigue necessary for these adverse outcomes remains to be established. Evidence also suggests that individuals' reasoning about their constitutional protections may be impaired, but more work is needed. Future studies should conduct more robust tests of how sleep loss and circadian misalignment impact eyewittness recognition, obedience to authority, and comprehension of legal risks as well as constitutional rights. Finally, there is a lack of evidence regarding how the public and potential jurors view the impact of sleep deprivation on legal decision-making or voluntariness of criminal admissions. We hope that this brief review provides useful guideposts while exposing gaps that should motivate further research.

## Data availability statement

The original contributions presented in the study are included in the article, further inquiries can be directed to the corresponding author.

## Author contributions

ZK: Conceptualization, Funding acquisition, Methodology, Writing – original draft, Writing – review & editing. BC: Writing – review & editing.
